# The Prognostic and Predictive Role of Chromogranin A in Gastroenteropancreatic Neuroendocrine Tumors – A Single-Center Experience

**DOI:** 10.3389/fonc.2021.741096

**Published:** 2021-11-12

**Authors:** Hui-Jen Tsai, Chin-Fu Hsiao, Jeffrey S. Chang, Li-Tzong Chen, Ying-Jui Chao, Chia-Ju Yen, Yan-Shen Shan

**Affiliations:** ^1^ National Institute of Cancer Research, National Health Research Institutes, Tainan, Taiwan; ^2^ Department of Oncology, National Cheng Kung University Hospital, College of Medicine, National Cheng Kung University, Tainan, Taiwan; ^3^ Department of Internal Medicine, Kaohsiung Medical University Hospital, Kaohsiung, Taiwan; ^4^ Institute of Population Health Sciences, National Health Research Institutes, Zhunan, Taiwan; ^5^ Division of General Surgery, Department of Surgery, National Cheng Kung University Hospital, College of Medicine, National Cheng Kung University, Tainan, Taiwan; ^6^ Institute of Clinical Medicine, College of Medicine, National Cheng Kung University, Tainan, Taiwan

**Keywords:** chromogranin A, neuroendocrine tumors, gastroenteropancreas, prognostic factor, predictive factor

## Abstract

Chromogranin A (CgA) is a non-specific biomarker excreted by neuroendocrine tumor (NET) cells. Elevation of circulating CgA level can be detected in gastroenteropancreatic (GEP)-NET patients and has been shown to correlate with tumor burden. The prognostic and predictive roles of CgA level and the change of CgA level are controversial. In this study, we retrospectively analyzed 102 grade 1/2 GEP-NET patients with available baseline or serial follow-up CgA levels from the National Cheng Kung University Hospital to evaluate the association between circulating CgA level and the tumor extent, overall survival (OS), and tumor response prediction. The baseline characteristics, baseline CgA level, and change of CgA level during follow-up and their association was analyzed. Sixty cases had baseline CgA levels available prior to any treatment and ninety-four cases had serial follow-up CgA levels available during treatment or surveillance. Baseline CgA levels were associated with stage and sex. Higher baseline CgA levels were associated with worse OS after adjusting for sex, stage, grade, primary site, and functionality (hazard ratio=13.52, 95% confidence interval (CI), 1.06-172.47, *P*=0.045). The cross-sectional analysis for the change of CgA level during follow-up showed that a ≥ 40% increase of CgA meant a higher probability of developing tumor progression or recurrence than those with a < 40% increase of CgA level (odds ratio=5.04, 95% CI, 1.31-19.4, *P*=0.019) after adjusting for sex, age, grade, stage, and functionality. Our study results suggest that CgA may be a predictive marker for tumor burden, OS, and tumor progression in GEP-NET patients.

## Introduction

Neuroendocrine tumors (NETs) are relatively rare neoplasms with neuroendocrine differentiation. NETs may arise from anywhere in the body with gastroentero-pancreatic (GEP) sites being the most common origins of NETs (1–3). The incidence of NETs has been increasing rapidly in recent decades (1–5). NETs are divided into two categories, functioning and non-functioning tumors. Functioning tumors secret substances causing specific symptoms. The substances produced by non-functioning tumors do not cause specific symptoms (6). The presence of specific symptoms may facilitate early diagnosis of NETs. For example, NETs excreting insulin (insulinoma) may induce hypoglycemia and cause dizziness, weakness, sweating, or consciousness disturbance that can be easily identified and diagnosed at an early stage. However, early diagnosis of non-functioning tumors is difficult due to the lack of specific symptoms. Because NETs are of a heterogeneous nature, the secreted substances differ among different NETs. The specific substances secreted by functioning GEP-NETs include insulin, glucagon, gastrin, serotonin, somatostatin, and vasoactive intestinal peptide (6). However, most tumor cells produce non-specific substances, such as chromogranin A (CgA) and neuro-specific enolase (NSE). These circulating biomarkers may play a diagnostic, prognostic, or predictive role for GEP-NETs; however, there are also limitations noted for these biomarkers (6).

CgA is a member of granins, which are abundantly distributed in endocrine, neuroendocrine, and immune cells. CgA can be proteolytically cleaved into biologically active peptides, such as vasostatin, pancreastatin, catestatin, and serpinins, by various enzymes, such as prohormone convertases, cathepsin L, plasmin, and kallikrein. CgA and its fragments can be detected in the blood of patients of various non-cancer diseases, such as heart failure, hypertension, thyroid disease, renal failure, liver disease, inflammatory bowel disease, rheumatoid disease, and cancers. They may also play roles in cardiovascular, immunometabolic, and cancer regulation (7–9). CgA is the most commonly used circulating biomarker for NETs in clinical practice. The sensitivity of circulating CgA is considered acceptable for the diagnosis of functional and advanced NETs whereas the specificity of circulating CgA for the diagnosis of NETs is not ideal. In addition to non-cancer disease, many other factors may interfere with the CgA level, such as food and drugs. CgA level can also be elevated in other non-NET cancers, such as breast cancer, thyroid cancer, pancreatic cancer, hepatocellular carcinoma, gastric cancer, colon cancer, and prostate cancer (8, 9). Therefore, the use of circulating CgA in diagnosis or screening has been limited. Nevertheless, circulating CgA has been commonly used for the follow-up of NETs (7–9). CgA level has been associated with the disease extent of GEP-NETs (10–12). Circulating CgA has been shown to be a biomarker for predicting survival and treatment response in advanced NET patients, but fewer studies were conducted for early-stage NETs (13–16). We retrospectively identified grade 1 (G1) and grade 2 (G2) GEP-NET patients with available baseline CgA or with serial follow-up CgA levels to correlate CgA levels with the clinical characteristics and outcomes.

## Patients and Methods

### Patient Identification and Data Collection

Patients diagnosed with G1 or G2 GEP-NETs at the National Cheng Kung University Hospital and with CgA levels available were included in this study. The data of patients’ demographic characteristics, including sex and age at diagnosis, and clinical information, including grade, stage, primary site, and functionality of the tumor, baseline CgA levels, serial follow-up of CgA levels during treatment or surveillance, treatment response, and survival status were collected by chart review. The study protocol was reviewed and approved by the Institutional Review Board of National Cheng Kung University Hospital. Because this study is a retrospective review of the chart, no informed consent is needed.

### Evaluation of Response and Survival

The patients received serial image examination for evaluation of disease status. The advanced-stage patients who received systemic treatment had serial image follow-ups every 2 to 6 months. The early-stage patients who had curative resection of the tumors had serial image follow-ups every 3-12 months. Tumor response was evaluated retrospectively according to the Response Evaluation Criteria In Solid Tumors (RECIST) v1.1. Overall survival (OS) was defined as the time from the diagnosed date to death or the last follow-up date.

### Evaluation of CgA level

The CgA levels were measured every 1-12 months for the patients. The frequency of CgA measurement was determined by the disease status and duration of each visit. The measurement frequency of CgA levels was every 1 to 3 months for the advanced-stage patients under treatment. The measurement frequency of CgA levels was every 3 to 12 months for the patients who had received curative resection without residual disease. The blood samples were sent to the Union Clinical Laboratory (Taipei, Taiwan) for the measurement of CgA levels. An automated immunofluorescent assay was used to detect the CgA levels by using Kryptor, Brahms. The baseline CgA level was defined as the CgA level detected prior to any treatment of NET. A CgA level more than 2-fold the upper normal limit of CgA (101.9 ng/ml) was defined as high whereas a CgA level less than 2-fold the upper normal limit of CgA was defined as low. The change of CgA level (ΔCgA) was determined by subtracting the first CgA level detected prior to or during the treatment of NET from the last CgA level detected prior to the disease progression or recurrence. The value of ΔCgA divided by the first CgA level detected prior to or during the treatment of NET was defined as the ratio of change of CgA.

### Statistical Analysis

All statistical analyses were performed using SAS statistical software (Version 8.2, SAS Institute Inc., Cary, NC, U.S.A). Summary statistics, including mean and standard deviation, were provided for continuous variables. Frequencies and proportions were used to summarize categorical data. The differences between high and low baseline CgA levels in NET patients were analyzed by Pearson’s chi-square test or exact test for variables including sex, grade, functionality, stage, and primary site. The relationship between OS and the potential explanatory factors was determined using the Cox proportional hazards model. In addition, the survival probabilities were all estimated by the Kaplan-Meier method for each group. Logistic regression analyses were performed to assess the effects of variables on the risk of progressive disease (PD) events. All tests were two-tailed. A p value <0.05 was considered significant.

## Results

### Patient Characteristics

There were 102 patients diagnosed with G1 or G2 GEP-NETs at the National Cheng Kung University Hospital from 2008 to 2020 with baseline CgA or serial follow-up CgA levels available. The patient characteristics are listed in **Table 1**. The mean age of all patients was 53.7 (range: 18-82) years old. There were 55 (53.9%) men and 47 (46.1%) women. Sixty-three (61.8%) cases were G1 and thirty-nine (38.2%) cases were G2. The percentages of stages I, II, III, and IV of the cases were 47.1%, 17.6%, 7.8%, and 27.5%, respectively. According to the site, 68, 9, 8, and 7 cases were located in the pancreas, stomach, rectum, and duodenum, respectively. The other cases included NETs of ampulla of Vater (N=4), liver (N=3), colon (N=2), and appendix (N=1). Thirty (29.4%) cases were functioning tumors. Among these 102 cases, 60 cases had baseline CgA levels available prior to any treatment and 94 cases had serial follow-up of CgA levels available before and after treatment for further analysis.

**Table 1:** Baseline characteristics.

	N=102 (%)
Age at diagnosis	
Mean ± std	53.7 ± 14.3
Median, range	54, 18-82
Sex	
F	47 (46.1%)
M	55 (53.9%)
Grade	
G1	63 (61.8%)
G2	39 (38.2%)
Stage	
I	48 (47.1%)
II	18 (17.6%)
III	8 (7.8%)
IV	28 (27.5%)
Primary site	
Pancreas	68 (66.7%)
Non-pancreas	34 (33.3%)
Stomach	9
Rectum	8
Duodenum	7
Ampulla of Vater	4
Liver, colon, appendix	3, 2, 1
Functionality	
No	72 (70.6%)
Yes	30 (29.4%)

### Baseline CgA Level Was Associated With OS of NET Patients

The distributions of the 60 cases with available baseline CgA levels by various variables, including sex, primary site, grade, stage, and functionality of the tumor are shown in **Table 2**. The high or low baseline CgA levels in NET patients were not associated with the primary site, grade, and functionality of the tumors. A higher proportion of women had high baseline CgA levels than the men in our NET patients (*P*=0.045). The distribution of baseline CgA levels was associated with stage (*P*=0.023). Stage I patients had a lower percentage with high baseline CgA levels (low versus high, 21:6) than stage IV patients (low versus high, 12:10).

**Table 2:** The distribution of baseline CgA level in GEP-NETs.

	Low	High	P* value
	N = 38	N = 22	
Sex			0.045
Men	24 (63.2%)	8 (36.4%)	
Women	14 (37.8%)	14 (63.6%)	
Grade			0.229
G1	25 (65.8%)	11 (50%)	
G2	13 (34.2%)	11 (50%)	
Functionality			0.428
Y	15 (39.5%)	11 (50%)	
N	23 (60.5%)	11 (50%)	
Stage			0.023
I	21 (55.3%)	6 (27.3%)	
II	1 (2.6%)	5 (22.7%)	
III	4 (10.5%)	1 (4.5%)	
IV	12 (31.6%)	10 (45.5%)	
Primary site			0.294
Pancreas	29 (76.3%)	14 (63.6%)	
Non-pancreas	9 (23.7%)	8 (36.4%)	

*Chi-square test or exact test.

**Figure 1:**
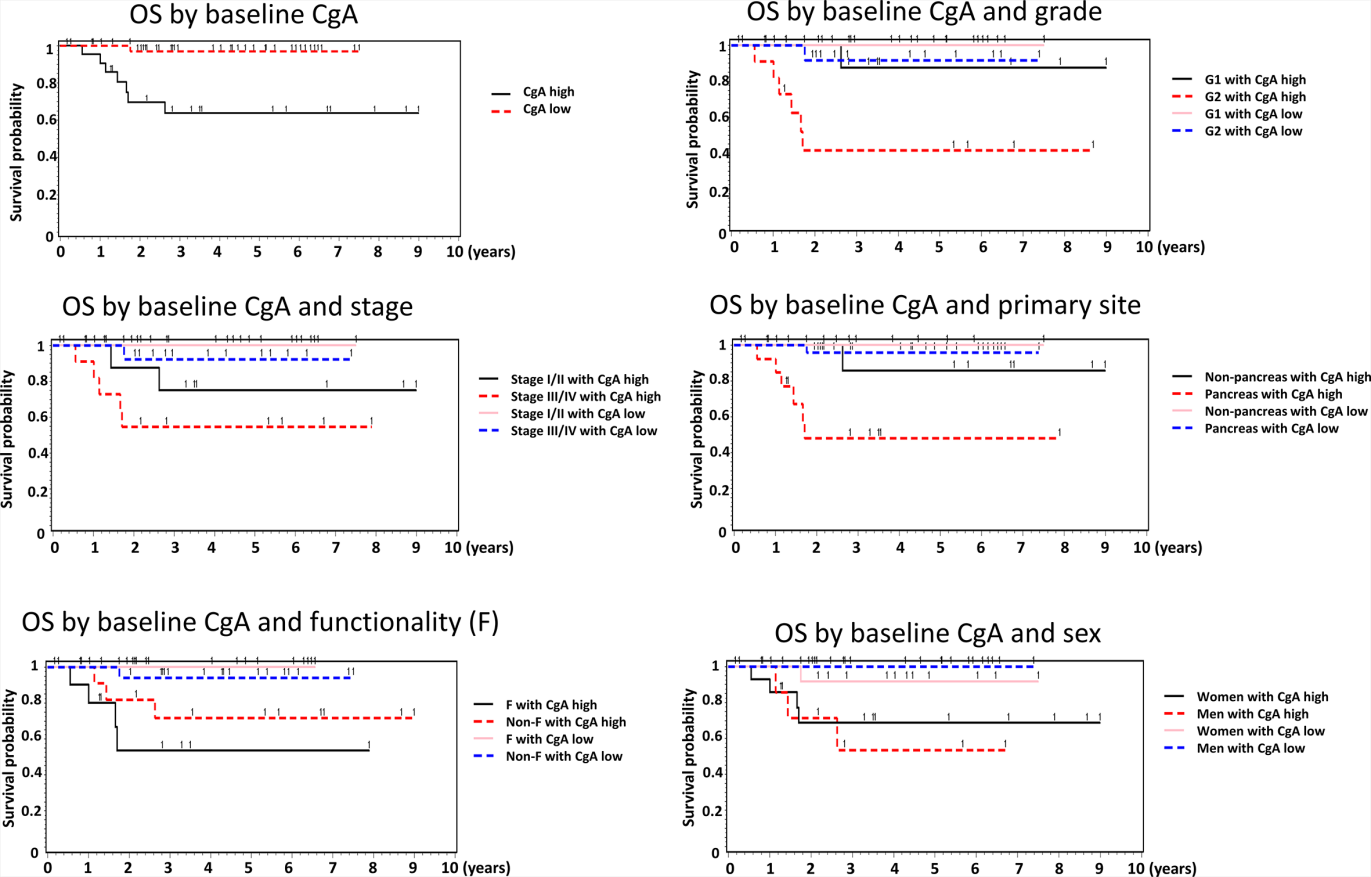
The Kaplan Meier survival curves of the GEP-NET patients by baseline CgA level in the overall patients and by sex, grade, stage, primary site, and functionality of the tumors.

We analyzed the association between baseline CgA levels and the OS of the NET patients (**Table 3**). The Kaplan Meier survival curves of the patients are shown in **Figure 1**. The OS was better in the patients with low baseline CgA levels. The survival rate in the patients with low baseline CgA levels was 97.4% whereas the survival rate in the patients with high baseline CgA levels was 68.2% (*P*=0.001). We further analyzed the association between OS and baseline CgA level by sex, primary site, grade, stage, and functionality of the tumors. The significant association between OS and baseline CgA levels in subgroup analysis was still present as shown in **Table 3**. Men with low baseline CgA levels had better OS than men with high baseline CgA levels (survival rate 100% vs 62.5%, *P*=0.001). The difference was not observed in women (survival rate 92.9% vs 71.4%, *P*=0.126). The patients with low baseline CgA levels had significantly better OS in pancreatic NETs (survival rate 96.6% vs 57.1%, *P*=0.0002) but not in non-pancreatic NETs (survival rate 100% vs 87.9%, *P*=0.317). The OS was significantly better in G2 (survival rate 92.3% vs 45.5%, *P*=0.007) patients with low baseline CgA levels than those with high baseline CgA level but the difference was not observed in G1 (survival rate 100% vs 90.9%, *P*=0.145) patients. The OS was high in G1 patients irrelevant of the baseline CgA levels. The OS was better for patients with functioning tumors who had low baseline CgA levels than those with high baseline CgA levels (survival rate 100% vs 63.6%, *P*=0.004). However, the OS was not significantly different in the NET patients with non-functioning tumors irrespective of their baseline CgA levels (*P*=0.098). The association between OS and the baseline CgA levels was significantly different in patients with stage I/II or III/IV tumors. The OS was better for the patients with stage I/II tumors who had low baseline CgA levels than those with high baseline CgA levels (survival rate 100% vs 81.8%, *P*=0.034). The survival rate of the stage III/IV patients with low baseline CgA levels was 93.8% but the survival rate of those with high baseline CgA levels was only 54.6% (*P*=0.019). The Cox proportional hazard ratio analysis for OS was performed by baseline CgA level, sex, age, grade, stage, and functionality (**Table 4**). Because there were fewer case numbers in stage II and III, we analyzed stage I and II combined versus stage III and IV combined. In the univariate analysis, patients with high baseline CgA levels had a worse OS than those with low baseline CgA levels (hazard ratio (HR) =14.31, 95% confidence interval (CI): 1.76-116.48). The G2 patients had a worse OS than those of G1 (HR = 11.71, 95% CI: 1.44-95.39). In multivariate analysis, significantly worse OS persisted for the patients with high baseline CgA levels versus low baseline CgA levels (HR = 13.52, 95% CI: 1.06-172.47) and for the patients of G2 NET versus G1 NET (HR = 41.81, 95% CI: 1.68-1041.72). The OS of the GEP-NET patients did not differ by age, sex, stage, and functionality.

**Table 3:** The overall survival rate of NET patients with low or high baseline CgA levels.

Baseline CgA level	Case number (N)	Survival rate (%)	Median survival (years)	P* value
All				0.001
Low	38	97.4	–	
High	22	68.2	–	
Baseline CgA level by sex				0.009
Men	32			0.001
Low	24	100	–	
High	8	62.5	–	
Women	28			0.126
Low	14	92.9	–	
High	14	71.4	–	
Baseline CgA level by primary site				0.0002
Pancreas	43			0.0002
Low	29	96.6	–	
High	14	57.1	–	
Non-pancreas	17			0.317
Low	9	100	–	
High	8	87.9	–	
Baseline CgA level by grade				<0.0001
G1	36			0.145
Low	25	100	–	
High	11	90.9	–	
G2	24			0.007
Low	13	92.3	–	
High	11	45.5	1.7	
Baseline CgA level by stage				0.002
I/II	33			0.034
Low	22	100	–	
High	11	81.8	–	
III/IV	27			0.019
Low	16	93.8	–	
High	11	54.6	–	
Baseline CgA level by functionality				0.004
Functioning	26			0.004
Low	15	100	–	
High	11	63.6	–	
Non-functioning	34			0.098
Low	23	95.7	–	
High	11	72.7	–	

*Log-rank test.

**Table 4:** The Cox proportional analysis for OS of NETs by baseline CgA level, sex, age, grade, stage, and functionality.

	Univariate		P* value	Multivariate		P* value
	HR	95% CI		HR	95% CI	
Baseline CgA level, high *vs* low	14.3	1.76-116.48	0.013	13.52	1.06-172.47	0.045
Age of diagnosis	1.04	0.99-10.9	0.160	1.08	0.98-1.19	0.101
Sex, men *vs* women	0.51	0.12-2.14	0.358	2.07	0.24-17.83	0.507
Grade, G2 *vs* G1	11.71	1.44-95.39	0.022	41.81	1.68-1041.72	0.023
Stage, III/VI *vs* I/II	3.86	0.78-19.16	0.098	3.66	0.33-40.10	0.288
Functionality, yes *vs* no	1.37	0.34-5.49	0.656	1.61	0.20-12.94	0.656

*Cox proportional analysis.

### The Change of CgA Level May Be a Predictor for Tumor Progression

Ninety-four cases had serial follow-up of CgA levels available during the follow-up. The ΔCgA for each patient was calculated. For the patients without PD, including complete response, partial response or stable disease, or without tumor recurrence, the ΔCgA was the value of the last CgA level detected prior to the last evaluation of tumor response subtracted by the first CgA level before or during treatment or surveillance of NET. The ratio of ΔCgA divided by the first CgA level before or during treatment or surveillance of NET was calculated. The cut off value of the ratio of change of CgA levels in the NET patients was 0.4 based on the significance of correlation with PD events (17). Specifically, logistic regression was performed to evaluate the correlation between the dichotomized variable and PD event. The optimal cut off was determined as the point with the most significant split. An increase of CgA level greater than 40% of the CgA level in the follow-up after treatment may predict tumor progression (**Table 5**). Patients with a change of CgA level greater than a 40% increase had a higher risk of tumor progression or recurrence than those with a change of CgA level less than a 40% increase (OR= 3.22, 95% CI: 1.11-9.34 in the univariate analysis; OR = 5.04, 95% CI: 1.31-19.4 in the multivariate analysis, adjusted for the age of diagnosis, grade, stage, functionality, and sex).

**Table 5:** Logistic regression analysis for odds ratio of tumor progression by ratio of change of CgA level, sex, age, grade, stage, and functionality.

	Univariate			Multivariate		
	OR	95% CI	*P* value	OR	95% CI	*P* value
Ratio of change of CgA level, ≥0.4 *vs* <0.4	3.22	1.11-9.34	0.031	5.04	1.31-19.4	0.019
Sex, men *vs* women	1.59	0.64-3.93	0.315	0.89	0.28-2.87	0.843
Age of diagnosis	0.98	0.95-1.01	0.217	1	0.96-1.04	0.950
Grade, G2 *vs* G1	8.4	3.02-23.34	<0.001	7.44	2.19-25.28	0.001
Stage, III/IV *vs* I/II	5	1.92-13.0	0.001	3.15	0.96-10.31	0.058
Functionality, yes *vs* no	0.82	0.3-2.25	0.704	0.49	0.13-1.82	0.288

## Discussion

Our study showed that high baseline CgA levels were associated with advanced stage in GEP-NETs. High baseline CgA levels and G2 were independent factors to predict the poor OS for GEP-NET patients. A 40% or greater increase of CgA level during follow-up might be a predictive factor for tumor progression or recurrence of GEP-NETs.

CgA level has been shown to be elevated in various diseases, including benign and malignant diseases. Mild elevation of CgA level has been shown in a variety of systemic diseases, such as cardiovascular disease (hypertension, congestive heart failure, myocardial infarction), renal disease (renal failure), liver disease (liver dysfunction, liver cirrhosis), lung disease (chronic obstructive pulmonary disease), inflammatory diseases (inflammatory bowel disease, rheumatoid arthritis), and sepsis (8). Mild elevation of CgA has also been detected in some benign or malignant cancers, such as parathyroid adenoma, thyroid cancer, hepatocellular carcinoma, and lung cancer (small cell carcinoma and non-small cell carcinoma) (8). Marked elevation of CgA was noted in neuroendocrine tumors (8). Elevation of CgA can also be related to acid suppressive medications (9). The non-oncologic and non-NET oncologic conditions impair the specificity of CgA for the diagnosis or the prognosis of NETs. NETest, a PCR-based 51-transcript signature for NETs, has been shown to have a higher sensitivity and specificity than CgA for the detection of NETs or prediction of tumor progression (18–20). However, it is still not routinely used in clinical practice probably due to cost and technical concerns. Although the specificity of CgA is not good, CgA is the most common non-specific circulating biomarker used for the follow-up of neuroendocrine tumors.

The sensitivity of CgA was reported to be related to the functionality and the extent of the NETs. Nehar et al. have shown that the sensitivity in patients with secreting tumors and non-secreting tumors was 73% and 45%, respectively (P <0.004) at the cut off value of 130 ng/ml of CgA. Significantly higher levels of CgA were noted in patients with metastatic disease (3444±16256 ng/ml) than those without (174±233 ng/ml, *P*<0.001) (11). Campana et al. have also shown that CgA levels were higher in NET patients than those with chronic active gastritis or healthy participants (12). Higher levels of CgA were observed in NET patients with diffuse disease compared to those with local or hepatic disease, and those that were disease-free (12). Jason et al. have shown that the CgA levels were significantly higher in GEP-NET patients with more than five liver metastases than those with fewer than five liver metastases or lymph node metastases (10). Advanced age and a CgA level greater than 5000 ng/ml were independent prognostic factors for worse OS in midgut NETs (10). Raoof et al. have reported that 79% of 445 small pancreatic NET (≤2 cm) patients were categorized with low CgA at a cut off value of 420 ng/ml (21). In our study, 21 of the 27 stage I patients (77.8%) had low baseline CgA levels whereas 12 of the 22 stage IV patients (54.5%) had low baseline CgA levels. Our result also showed an association between the baseline CgA levels and the extent of NETs and this is compatible with the findings of the previous studies. However, we did not observe any differences in the baseline CgA levels in GEP-NETs by grade (G1 vs G2), functionality, or primary site (pancreas vs non-pancreas) in our study. Different from other studies, we observed that lower baseline CgA levels were noted in men (75%) than in women (50%, *P*=0.045).

The CgA level was also reported to be associated with the prognosis of NET patients. Yao et al. analyzed and reported the prognostic role of CgA and NSE in patients with low- to intermediate-grade advanced pancreatic NET from the RADIANT-1 phase II study. Elevated baseline CgA levels were associated with a shorter progression-free survival (PFS, 8.34 months in the elevated CgA group vs 15.64 months in the non-elevated CgA group, *P*=0.03) and OS (16.95 months vs not reached, *P <*0.001). The prognostic role was also observed in NSE (14). Yao et al. also evaluated the impact of several biomarkers (baseline levels of CgA, NSE, and multiple soluble angiogenetic biomarkers) on the OS of advanced, progressive, low- or intermediate-grade pancreatic NET patients who received everolimus or placebo in the RADIANT-3 trial. The lower baselines of CgA, NSE, placental growth factor (PIGF), and soluble vascular endothelial growth factors 1 were associated with better OS. However, only NSE and PIGF remained significantly associated with OS in the multivariate analysis. The effect of CgA was borderline significant (HR=0.76, 95% CI, 0.57-1, *P*=0.05) in the multivariate analysis (13). Ahmed et al. analyzed the data of 360 patients with midgut NETs with liver metastases from the UKI NET group. They reported that increasing age at diagnosis, higher Ki-67, increasing urinary hydroxyindole acetic acid levels, higher CgA levels, high tumor volume, and resection of primary tumor were associated with a worse OS in the univariate analysis (22). However, only age, Ki-67, and resection of primary tumor were identified as the independent predictors of survival in multivariate analysis (22). Chou et al. have reported that Eastern Cooperative Oncology Groups performance score 0-1, G1-2, single organ metastasis, and baseline CgA level less than twice the upper normal range were independent prognostic factors for OS of advanced GEP-NET patients (15). Most studies demonstrated the prognostic role of CgA in advanced GEP-NETs whereas some studies showed no significant role of CgA as the predictor for OS. Fewer studies analyzed the prognostic role of CgA level in early-stage cases. Raoof et al. showed that CgA level (high vs low at a cut off value of 420 ng/ml) was an independent predictive factor for OS in small pancreatic NET (tumor ≤ 2 cm) patients in multivariate analysis after adjusting for tumor size, grade, nodal status, and academic status of the facility (HR=7.9, 95% CI, 2.34-26.69, *P*=0.001) (21). They also observed that the OS was not significantly different between patients with low CgA levels receiving or not receiving tumor resection. But the OS was worse for the patients with high CgA levels who had not received tumor resection than those who had received tumor resection.^21^ In our study, we observed that the patients with low baseline CgA levels had significantly better OS than those with high baseline CgA levels. The significance persisted in multivariate analysis after adjusting for age, sex, grade, stage, and functionality. The result supported the prognostic role of baseline CgA levels in GEP-NETs, including early-stage and advanced-stage patients.

Circulating CgA during the follow-up of GEP-NET patients receiving systemic treatment or surveillance has also been investigated. Yao et al. reported that early CgA and NSE response (≥ 30% decrease of CgA or NSE from baseline or normalization at week 4) were predictors for longer PFS and OS in advanced pancreatic NET patients receiving everolimus treatment (RADIANT-1 study). The median PFS for the patients with early CgA response was 13.31 months whereas the median PFS for those without early response was only 7.52 months (*P*<0.001). The median OS for the patients with an early CgA response was 24.9 months whereas the median OS for those without an early CgA response was 12.71 months (*P*=0.01) (14). Jensen et al. retrospectively analyzed the change of CgA level during treatment for CgA-producing ileo-cecal NET patients. They demonstrated a cut off of 25% for the prediction of tumor response after treatment with a 25% or greater increase predicting tumor progression and a 25% or greater decrease predicting tumor regression (23). Chou et al. also analyzed the change of CgA level and tumor response for advanced GEP-NET patients during treatment. They demonstrated that a change of CgA level >17% distinguished partial response and stable disease from progressive disease with a sensitivity and specificity of 91.2% and 82.9%, respectively (15). However, some studies did not validate CgA as a surrogate marker for tumor progression of NETs. Vezzosi et al. prospectively analyzed the concordance between CgA variation and RECIST criteria for tumor response in 39 metastatic well-differentiated GEP-NET patients. They showed that change of CgA at the 6-month follow-up (≥ 25% increase versus < 25% increase) had a sensitivity and a specificity of 71% and 50%, respectively, for changes of tumor burden. The study did not validate CgA as a surrogate marker of tumor progression (24). Dam et al. prospectively monitored CgA levels for GEP-NET patients with metastasis or residual tumors and analyzed the predictive role of change of CgA level for tumor progression and regression. They reported an overall Spearman’s rank correlation coefficient of 0.17 (*P*=0.003) by analyzing the “matching pairs” of CgA and CT/MRI assessment. The diagnostic sensitivity and specificity of an increased CgA level for tumor progression were 36% and 82%, respectively. The diagnostic sensitivity and specificity of a decreased CgA level for tumor regression were 79% and 69%, respectively. They concluded a weak association between change of CgA and change in tumor burden (25). In our study, we observed that the patients with a 40% or greater increase of CgA during treatment or surveillance had a higher risk of developing tumor progression than those with less than a 40% increase of CgA during follow-up with an OR of 5.04 (95% CI, 1.31-19.4, *P*=0.019) by multivariate logistic regression. The result supported the predictive role of change of CgA level for tumor progression in advanced GEP-NETs. Our patient population included early and advanced stages. The results suggested that an increase of CgA may also predict recurrence of early-stage GEP-NET after complete resection.

This is a retrospective study and the frequency and timing of measurement and follow-up of CgA level and tumor response varied according to their disease status and treatment. Therefore, we could not use repeated measurements of CgA to evaluate longitudinal change of CgA for tumor response prediction. However, we used cross-sectional analysis for the change of CgA to predict tumor progression that was not interfered with by the fluctuation of CgA level during serial follow-up. Furthermore, to gain acceptance as a clinically meaningful observation, the change of CgA level would require testing in multi-center clinical therapeutic trials. Most importantly, the method of matching should be performed to reduce or eliminate the effects of confounding.

## Conclusions

Our results suggested that baseline CgA level is associated with the disease extent and OS of GEP-NET patients. A 40% or greater increase of change of CgA level may predict tumor progression or recurrence during treatment or surveillance of GEP-NETs.

## Data Availability Statement

The datasets presented in this article are not readily available because the dataset is not available due to IRB restriction. Requests to access the datasets should be directed to Y-SS, ysshan@ncku.edu.tw.

## Ethics Statement

The studies involving human participants were reviewed and approved by the Institutional Review Board of National Cheng Kung University Hospital. Written informed consent for participation was not required for this study in accordance with the national legislation and the institutional requirements.

## Author Contributions

H-JT had acquisition of data, analyzed and interpreted data, and wrote and revised the manuscript. C-FH analyzed and interpreted the data and wrote and revised the manuscript. JC analyzed and interpreted the data and revised the manuscript. L-TC, Y-JC, and C-JY analyzed and interpreted the data and revised the manuscript. Y-SS designed the study, analyzed and interpreted the data and revised the manuscript. All authors had final approval of the version of the manuscript to be submitted.

## Conflict of Interest

The authors declare that the research was conducted in the absence of any commercial or financial relationships that could be construed as a potential conflict of interest.

## Publisher’s Note

All claims expressed in this article are solely those of the authors and do not necessarily represent those of their affiliated organizations, or those of the publisher, the editors and the reviewers. Any product that may be evaluated in this article, or claim that may be made by its manufacturer, is not guaranteed or endorsed by the publisher.
